# Rapid, Large, and Synchronous Sweat and Cardiovascular Responses Upon Minor Stimuli in Healthy Subjects. Dynamics and Reproducibility

**DOI:** 10.3389/fneur.2020.00051

**Published:** 2020-02-04

**Authors:** Ai Van Thuy Ho, Karin Toska, Jarlis Wesche

**Affiliations:** ^1^Faculty of Medicine, Institute of Clinical Medicine, University of Oslo, Oslo, Norway; ^2^Department of Vascular and Thoracic Surgery, Akershus University Hospital, Lørenskog, Norway; ^3^Department of Physiology, Institute of Basic Medical Sciences, University of Oslo, Oslo, Norway; ^4^Department of Medical Biochemistry, Oslo University Hospital, Oslo, Norway

**Keywords:** electrodermal activity, skin blood flow, mental challenge, deep inspiration, heart rate, radial artery blood flow

## Abstract

**Purpose:** The aim of the study was to investigate steady state levels, dynamics and reproducibility of cardiovascular variables and electrodermal activity in different skin areas in response to minor physiological and mental stimuli in healthy subjects in the thermoneutral zone, carried out in high time resolution.

**Methods:** Thirteen healthy subjects underwent experiments on two separate days. Non-invasive electrodermal activity in five different skin areas was measured continuously using a skin conductance method, including resting supine and sitting positions, performing deep inspirations, a mental challenge and being exposed to a sudden loud sound. Blood pressure, heart rate, radial artery blood flow, and skin perfusion were measured simultaneously.

**Results:** Electrodermal activity in the right and left palms was almost identical, with rapid and large increases within a few seconds in response to stimuli, whereas no such significant changes were seen in the face, back, and abdomen. Radial artery blood flow and palmar skin perfusion changed synchronously with electrodermal activity for each stimulus, and were correlated to changes in blood pressure and heart rate. The response patterns in each subject were very similar on the two experimental days. There was very low spontaneous electrodermal activity in the supine position, contrary to the resting sitting position.

**Conclusion:** The electrodermal activity increased rapidly and synchronously in both palms within a few seconds as a response to minor physiological and mental stimuli, synchronous with fluctuations in radial artery blood flow, palmar skin perfusion, and cardiovascular variables. The responses are reproducible from day to day, making them a stable and constant stimuli to be used for studies in patients with hyperhidrosis.

## Introduction

Sweat glands are classified into apocrine and eccrine, although the mechanism of sweat secretion is probably the same (1–3). There are two types of sweating; thermoregulatory sweating, which occurs over the whole body in response to changes in central body temperature, and emotional sweating which is limited to the palms, axillae, and soles of the feet (3–7). Apocrine sweat glands are found mainly in the axillae, areola, and pubic and perianal regions (8, 9). Eccrine sweat glands are found on the glabrous skin of the palm and sole, where they are usually not activated by heat, but rather by deep inspiration, mental stress and local tactile stimulation (emotional sweating) (2, 7, 8). Synchronous sweat secretion was observed in the 1970's and identification of varied responses related to thermoregulation has been studied by Ogawa et al. (10–12). In the thermoneutral zone (which for naked resting humans is about 26–36°C) (13), no sweat activity is observed, and core body temperature is kept in the constant range by variations in blood flow to the body surface. These adjustments are mainly obtained by fluctuations in perfusion of acral skin and controlled by sympathetic autonomic nerves to specialized vascular structures of arteriovenous anastomosis (AVAs) (13–17). AVAs are mostly located in the nailbeds and glabrous skin of palms and feet and are densely innervated by sympathetic nerve fibers. There is also a close relationship between these AVA flow fluctuations, heart rate, and arterial pressure (13, 14, 17–19). In addition, stimuli eliciting sympathetic nerve responses, such as deep inspiration or fright, are known to cause increased electrodermal activity, unrelated to the thermoregulatory function of the sweat glands. There is a lack of knowledge, however, about the dynamics and reproducibility of electrodermal activity and its relation to acral blood flow and central cardiovascular responses in healthy humans in the thermoneutral zone.

Thus, we wanted to investigate the dynamics of electrodermal activity and cardiovascular changes in healthy humans in the thermoneutral zone when being exposed to minor mental and physiological stimuli, using a portable instrument to measure rapid and small changes in electrodermal activity (13, 14, 17–20). Such responses may reveal new aspects and details of the organization of autonomic nervous responses to such stimuli (21) and could be an important tool to diagnostic and prognostic evaluation of patients with hyperhidrosis who might be a candidate for surgery (thoracic sympathectomy).

## Methods

### Subjects

Thirteen healthy subjects, three males and 10 females, aged 19–52 years (mean age 25.8) and BMI (18.5–24.9) were included. They did not use any medication, except one woman who used birth control pills, and her sweat patterns did not differ from the other subjects. Participants were instructed to refrain from strenuous exercise, consumption of alcohol, tobacco, and caffeine for at least 12 h prior to the experiment. The recordings were conducted during 3 weeks in the summer season. All participants gave written informed consent and the study was approved by the regional ethics committee.

### Experimental Design

Measurements were obtained continuously over a period of 30 min; 10 min in supine (SUP) and 20 min in sitting position (SIP). During the last 10 min in the sitting position, the subjects were exposed to a series of stimuli starting by taking a deep inspiration three times every 1 min (INSP1, INSP2, INSP3), with normal respiration between the deep inspirations, followed by a mental challenge (MC) by performing a mental subtraction task (from 100 minus 7 each time) lasting 1 min, and then being subject to a sudden (< 0.5 s) sound stimulus (SS). A single hand clap behind the subject created a sound at ~90 dB. These stimuli are known to elicit physiological and emotional responses (12, 22–25).

**Figure 1:**
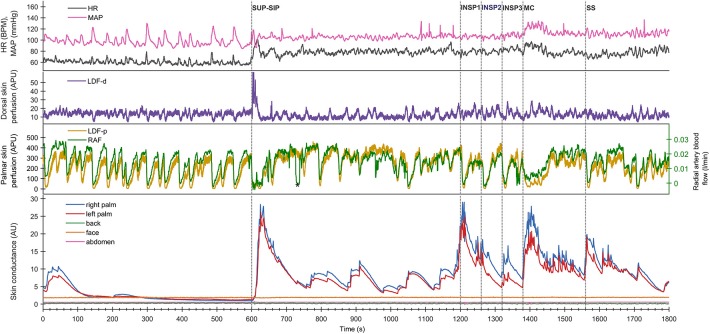
Shows simultaneous beat-to-beat recordings of MAP, HR, radial artery blood flow (RAF), palmar and dorsal hand skin perfusion (LDF-p and LDF-d), and electrodermal activity from five different skin areas in one healthy subject during a 30 min period. The color coding of the traces is explained to the left in the figure. The x-axis is time in seconds, while the y-axis is BPM for HR, mmHg for MAP, arbitrary perfusion units (APU) for laser Doppler for skin perfusion, L/min for radial artery blood flow and arbitrary units of skin conductance recorded in the right and left hypothenar region of the palm, back, face, and abdomen, respectively. Vertical dashed lines mark the start of different stimuli corresponding to position change from supine (SUP) to sitting (SIP) (SUP-SIP), three deep inspirations (INSP1-3), mental challenge (MC), and sound stimulus (SS).

**Figure 2:**
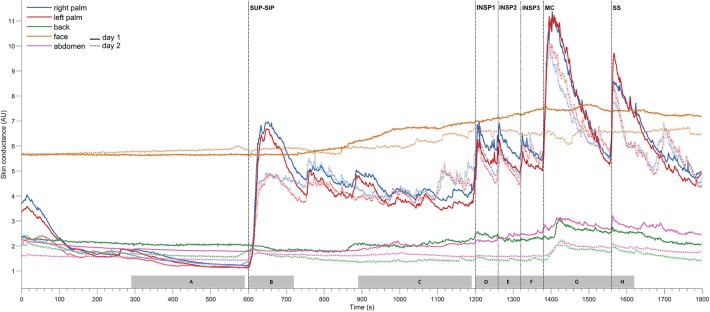
Shows mean of responses of electrodermal activity from 13 subjects marked with solid lines for the 1st experimental day (day 1), and dashed lines for the 2nd day (day 2), and shows simultaneous recordings from five different skin areas (color coding of the traces explained to the left in the figure). The x-axis is time in seconds, while the y-axis is skin conductance in arbitrary units. Vertical dashed lines mark the start of different stimuli corresponding to position change from supine (SUP) to sitting (SIP) (SUP-SIP), three deep inspirations (INSP1-3), mental challenge (MC) and a sound stimulus (SS). The gray boxes on the x-axis **(A–H)** shows the time periods from which the values for the different stimuli are calculated and shown in Table 1.

Subjects were asked to make themselves comfortable in the experimental setting and to refrain from moving during the entire experiment to avoid artifacts and biofeedback in the recordings, and to limit skin conductance changes due to physical activity. To further minimize the impact of experimental artifacts, participants were not allowed to look at the recording computer throughout the trial. During the change in body position from SUP to SIP the experimental leader helped and supported the subject to avoid wires being twisted and no extra muscle activity being carried out. A weak beeping sound was given from the computer 3 s before each stimulus, for the experimental leader to give timely instructions to the subjects. At the last beep, a marker was set in the recording file to indicate the start of each stimulus, marked as SUP-SIP, INSP1, INSP2, INSP3, MC, and SS by the vertical lines in the figures. The experimental protocol is illustrated in Figures 1, 2, **4**.

The subjects were lightly dressed, and the ambient temperature was kept at 27 ± 1°C to keep the subjects in the thermoneutral zone. Prior to the test, each subject received instruction on the different stimuli, and practiced the test. None reported discomfort or feeling cold or warm during the experiments. The subject was placed on a table in the supine position and allowed to rest for at least 30 min before each experimental session. Each subject underwent two separate days of measurement with 1–18 days between each experimental day.

### Measurements

A multichannel Sudologger® (BioGauge AS, Oslo Innovation Center, Oslo, Norway) was used for non-invasive simultaneous continuous measurements of electrodermal activity in three glabrous locations at both palms and face, and the remaining two non-glabrous locations at the back and abdomen (20). The Sudologger® uses skin surface electrodes for unipolar conductance measurements in the stratum corneum, and the method was introduced and described by Tronstad et al. (20).

Beat-to-beat radial artery blood flow (RAF) was recorded using ultrasound Doppler (SD-100, GE Vingmed Ultrasound, Horten, Norway) as described previously (19). The operating frequency was 10 MHz. A button-shaped transducer was attached with adhesive tape to the skin surface over the radial artery and the ultrasound beam was directed at the radial artery at the wrist level at a constant angle of 45°. Care was taken to make sure that the beam covered the whole cross-section of the vessel. Instantaneous intensity-weighted mean velocity was calculated by the velocimeter and interfaced online to a recording computer.

Laser Doppler Flux (LDF) (MBF3D, Moor Instruments, Devon, UK) was used to measure continuous skin perfusion from the left palmar (LDF-p) and dorsal hand (LDF-d). LDF is reflected off red blood cells 1–2 mm below the skin surface and is previously described in detail (14, 26). The laser Doppler probes were fixed to the skin with double-sided tape. The noise-limiting filter of the instrument was set at its highest level (21 kHz), and the emitted wavelength was 820 nm. The flux output signal was filtered with a time constant of 0.1 s and sent to the computer. The sampling frequency was 2 Hz. The subject was kept out of sight from looking at the screen displaying the variables during the experiment to avoid any biofeedback.

Heart rate (HR) was calculated over each R-R interval of the electrocardiogram (ECG). Separately, the diameter of the radial artery was measured by ultrasound imaging (model CFM-750, GE Vingmed Ultrasound, Horten, Norway). The diameter of the radial artery in the supine position was determined as the average obtained from three frozen-screen arterial diameter images as it has been shown that pulsatile diameter of radial arteries is very stable, and the area was calculated assuming the vessel was circular (17). Finger arterial pressure was recorded continuously and non-invasively from the third finger of the left hand (2300 Finapres BP monitor, Ohmeda, Madison, WI, USA) (27). The left hand was continuously supported on a table during supine position at heart level, while placed on the thigh during the sitting position. In SIP, the height difference between the hand and heart was compensated for when calculating the mean arterial pressure (MAP). Corrected MAP is shown in the figures.

### Data Analysis and Statistics

The cardiovascular variables were recorded and stored with a sampling frequency of 50 Hz in a stationary computer. Two Sudologger® units were used and recordings of electrodermal activity measurements were saved in Sudologger® program on a laptop. Cardiovascular and electrodermal activity data were synchronized for time. Analysis of data was performed off-line using customized computer programs and a commercial software package, MATLAB (The MathWorks, Inc., Natick, MA, USA). Variations in the recorded variables to the experimental session were partly eliminated by calculating the averaged response from the different sweat areas of the skin and cardiovascular variables from the 13 subjects (coherent averaging) (28). The correlation of electrodermal activity between the left and right palm was calculated by MATLAB (The MathWorks, Inc., Natick, MA, USA).

Based on our own pilot measurements, we expected an change in electrodermal activity in the palms parallel to radial arterial blood flow and skin perfusion in palmar hand during physiological and mental stimuli of at least 50%. A sample size of seven healthy subjects would have 80% power to detect a difference in means of 50% with a standard deviation of 0.46, using a two-sided paired sample *t*-test with α 0.05. For all subjects, the statistical significance of differences between sweat patterns and cardiovascular variables during the 1st and 2nd experimental days were analyzed using paired samples *t*-tests and the mean scores for electrodermal activity during three inspirations were analyzed using ANOVA with repeated measures with a Greenhouse-Geisser correction, using IBM SPSS Statistics 22 (IBM/SPSS Inc., Armonk, NY, USA). Values are expressed as means, standard error of the mean (SEM), confidence interval (CI) and differences considered statistically significant at *p* < 0.05 (Table 1, Table S1).

**Table 1:** Mean values of electrodermal activity from five skin areas.

**Day 1**				
**SIP_**2min**_–SUP_**5min**_**	**Mean**	**SEM**	**95% CI**	***p*-value (2-tailed)**
Right palm	4.10	1.06	1.79–6.42	0.002^*^
Left palm	3.80	0.99	1.64–5.96	0.002^*^
Back	−0.52	0.38	−1.36–0.31	0.198
Face	−0.53	0.52	−1.65–0.59	0.326
Abdomen	−0.02	0.05	−0.12–0.09	0.737
**SIP**_**5min**_**–SUP**_**5min**_	**Mean**	**SEM**	**95% CI**	***p*****-value (2-tailed)**
Right palm	2.76	0.79	1.04–4.47	0.004^*^
Left palm	2.50	0.73	0.92–4.08	0.005^*^
Back	−0.02	0.39	−0.87–0.83	0.965
Face	1.00	0.70	−0.52–2.52	0.176
Abdomen	0.19	0.26	−0.37–0.75	0.478
**INSP1—SIP**_**5min**_	**Mean**	**SEM**	**95% CI**	***p*****-value (2-tailed)**
Right palm	2.01	0.76	0.35–3.67	0.015^*^
Left palm	1.84	0.65	0.42–3.26	0.015^*^
Back	0.21	0.25	−0.32–0.75	0.405
Face	0.11	0.09	−0.08–0.30	0.232
Abdomen	0.11	0.08	−0.07–0.29	0.194
**INSP2—SIP**_**5min**_	**Mean**	**SEM**	**95% CI**	***p*****-value (2-tailed)**
Right palm	1.88	0.70	0.36–3.40	0.020^*^
Left palm	1.70	0.64	0.32–3.09	0.020^*^
Back	0.02	0.16	−0.32–0.37	0.879
Face	0.27	0.21	−0.19–0.73	0.224
Abdomen	0.29	0.25	−0.26–0.85	0.274
**INSP3—SIP**_**5min**_	**Mean**	**SEM**	**95% CI**	***p*****-value (2-tailed)**
Right palm	1.68	0.69	0.18–3.18	0.031^*^
Left palm	0.96	0.47	−0.05–1.98	0.030^*^
Back	0.06	0.19	−0.36–0.47	0.765
Face	0.46	0.34	−0.27–1.19	0.198
Abdomen	0.39	0.32	−0.31–1.10	0.247
**MC**_**3min**_**–SIP**_**5min**_	**Mean**	**SEM**	**95% CI**	***p*****-value (2-tailed)**
Right palm	3.93	1.05	1.65–6.21	0.003^*^
Left palm	4.38	1.14	1.90–6.87	0.002^*^
Back	0.41	0.41	−0.49–1.30	0.339
Face	0.58	0.41	−0.31–1.47	0.180
Abdomen	0.78	0.36	0.00–1.55	0.050
**SS**_**1min**_**–SIP**_**5min**_	**Mean**	**SEM**	**95% CI**	***p*****-value (2-tailed)**
Right palm	3.82	0.95	1.75–5.88	0.002^*^
Left palm	4.54	1.17	1.99–7.08	0.002^*^
Back	0.35	0.38	−0.47–1.16	0.375
Face	0.49	0.41	−0.40–1.38	0.256
Abdomen	0.78	0.43	−0.15–1.71	0.093
**Day 2**				
**SIP_**2min**_–SUP_**5min**_**	**Mean**	**SEM**	**95% CI**	***p*-value (2-tailed)**
Right palm	2.88	0.91	0.89–4.87	0.008^*^
Left palm	2.80	0.81	1.04–4.55	0.005^*^
Back	0.05	0.23	−0.46–0.56	0.825
Face	0.04	0.05	−0.07–0.15	0.471
Abdomen	0.19	0.20	−0.25–0.63	0.370
**SIP**_**5min**_**–SUP**_**5min**_	**Mean**	**SEM**	**95% CI**	***p*****-value (2-tailed)**
Right palm	2.92	1.00	0.75–5.09	0.012^*^
Left palm	2.90	0.85	1.05–4.75	0.005^*^
Back	−0.21	0.26	−0.78–0.36	0.440
Face	0.31	0.39	−0.55–1.16	0.449
Abdomen	0.16	0.26	−0.40–0.73	0.539
**INSP1—SIP**_**5min**_	**Mean**	**SEM**	**95% CI**	***p*****-value (2-tailed)**
Right palm	0.66	0.86	−1.20–2.52	0.455
Left palm	0.74	0.56	−0.48–1.97	0.210
Back	0.04	0.04	−0.04–0.12	0.278
Face	0.12	0.08	−0.06–0.30	0.162
Abdomen	0.02	0.03	−0.05–0.08	0.594
**INSP2—SIP**_**5min**_	**Mean**	**SEM**	**95% CI**	***p*****-value (2-tailed)**
Right palm	0.34	0.92	−1.66–2.35	0.715
Left palm	0.64	0.76	−1.01–2.29	0.413
Back	0.00	0.02	−0.04–0.05	0.948
Face	0.14	0.10	−0.07–0.36	0.167
Abdomen	0.01	0.05	−0.11–0.12	0.920
**INSP3—SIP**_**5min**_	**Mean**	**SEM**	**95% CI**	***p*****-value (2-tailed)**
Right palm	0.90	0.61	−0.42–2.22	0.162
Left palm	1.19	0.54	0.01–2.38	0.048
Back	0.01	0.02	−0.05–0.06	0.757
Face	0.01	0.22	−0.48–0.50	0.961
Abdomen	0.01	0.06	−0.12–0.14	0.855
**MC**_**3min**_**–SIP**_**5min**_	**Mean**	**SEM**	**95% CI**	**p-value (2-tailed)**
Right palm	2.25	0.91	0.26–4.24	0.030^*^
Left palm	3.09	0.73	1.50–4.69	0.001^*^
Back	0.41	0.38	−0.41–1.23	0.294
Face	0.01	0.08	−0.17–0.18	0.911
Abdomen	0.37	0.21	−0.08–0.81	0.101
**SS**_**1min**_**–SIP**_**5min**_	**Mean**	**SEM**	**95% CI**	***p*****-value (2-tailed)**
Right palm	1.80	0.79	0.07–3.53	0.043^*^
Left palm	2.53	0.70	1.00–4.06	0.004^*^
Back	0.38	0.42	−0.54–1.29	0.385
Face	0.10	0.12	−0.15–0.36	0.388
Abdomen	0.39	0.26	−0.19–0.96	0.169
**Day 1 vs. Day 2**				
**SUP–SUP**	**Mean**	**SEM**	**95% CI**	***p*-value (2-tailed)**
Right palm	1.32	1.34	−1.60–4.24	0.343
Left palm	1.09	1.29	−1.71–3.89	0.413
Back	−0.09	0.53	−1.26–1.07	0.865
Face	−0.75	1.17	−3.30–1.80	0.532
Abdomen	0.15	0.27	−0.44–0.74	0.604
**INSP1**	**Mean**	**SEM**	**95% CI**	***p*****-value (2-tailed)**
Right palm	0.51	1.49	−2.73–3.75	0.737
Left palm	0.34	1.26	−2.41–3.08	0.794
Back	0.95	0.75	−0.69–2.59	0.229
Face	0.46	1.16	−2.08–2.99	0.702
Abdomen	0.61	0.29	−0.03–1.24	0.061
**INSP2**	**Mean**	**SEM**	**95% CI**	***p*****-value (2-tailed)**
Right palm	0.70	1.32	−2.17–3.57	0.606
Left palm	0.30	1.22	−2.35–2.95	0.809
Back	0.80	0.62	−0.54–2.14	0.216
Face	0.59	1.21	2.04–3.22	0.632
Abdomen	0.80	0.40	−0.07–1.67	0.069
**INSP3**	**Mean**	**SEM**	**95% CI**	***p*****-value (2-tailed)**
Right palm	−0.06	1.26	−2.80–2.68	0.962
Left palm	−0.39	1.28	−3.17–2.39	0.766
Back	0.83	0.58	−0.44–2.10	0.180
Face	0.92	1.16	−1.61–3.44	0.446
Abdomen	0.89	0.46	−0.12–1.90	0.078
**MC**	**Mean**	**SEM**	**95% CI**	***p*****-value (2-tailed)**
Right palm	0.84	1.14	−1.66–3.34	0.477
Left palm	0.53	1.22	−2.14–3.19	0.673
Back	0.78	0.56	−0.44–1.99	0.188
Face	1.04	1.00	1.15–3.22	0.321
Abdomen	0.93	0.43	−0.01–1.87	0.053
**SS**	**Mean**	**SEM**	**95% CI**	***p*****-value (2-tailed)**
Right palm	1.19	0.95	−0.88–3.26	0.236
Left palm	1.25	0.93	−0.79–3.28	0.207
Back	0.75	0.58	−0.52–2.02	0.223
Face	0.85	1.08	−1.51–3.20	0.448
Abdomen	0.92	0.54	−0.27–2.11	0.117

*Mean values, SEM and 95% confidence interval (CI) from the right and left palm, back, face and abdomen from 13 subjects upon different stimuli as position change from supine (SUP) to sitting (SIP) position (SIP_2min_ – SUP_5min_ during 2 min and SIP_5min_ – SUP_5min_ during 5 min), INSP1 (1st inspiration during 1 min), INSP2 (2nd inspiration during 1 min), INSP3 (3rd inspiration during 1 min), MC (mental challenge during 3 min), and SS (sound stimulus during 1 min) from the 1st and 2nd day and comparison between these 2 days. These mean values are calculated from the time periods indicated by the gray boxes marked with A–H shown in Figure 2. Supine position values were calculated as the average during a 5 min period (SUP_5min_) (A), while values in the sitting position was calculated 2 min after the position change (SIP_2min_) (B), and 5 min later in the experiment, which represents the baseline values in the sitting position (SIP_5min_) (C). The time period marked with the gray box C represents the baseline for the 1st, 2nd, and 3rd inspirations (INSP1, INSP2, INSP3), mental challenge (MC) and sound stimulus (SS). Electrodermal activity during inspiration (three times) and sound stimulus were calculated as the average 1 min after each stimulus, whereas 3 min after mental challenge. Values are estimated paired t-test mean and 95% confidence intervals for the group of 13 healthy subjects*.

* *Significantly different from baseline (P < 0.05 for two-tailed test)*.

## Results

### Electrodermal Activity

Figure 1 (lower panel) shows a typical pattern of continuous recordings of electrodermal activity in the five different skin areas from one subject, at position change (from supine to sitting) (SUP-SIP), followed by three deep inspirations (INSP1-3), mental challenge (MC), and a sound stimulus (SS). Upon every stimulus, the electrodermal activity in both left and right palms increased rapidly and synchronously. No change in electrodermal activity was shown in the face, back, and abdomen. These patterns were seen in most of the subjects. The corresponding changes in cardiovascular variables are shown in the middle and upper panels and are described in detail below.

Figure 2 shows the mean of electrodermal activity responses from all 13 subjects, and shows that the electrodermal activity pattern was very similar on both experimental days. The peaks of electrodermal activity where different in magnitude in response to physiological and mental activity. Recordings in the palms showed immediate and large synchronous response upon every stimulus. The electrodermal activity response was largest after position change, mental challenge and sound stimulus. During position change, the electrodermal activity started to increase after 5 s with a slow and gradual response to its highest peak around 33 s after the stimulus. Furthermore, the electrodermal activity curve during mental challenge was almost identical to the curve of sound stimulus in the beginning, with a rapid and steep increase already 2 s after stimulus, and continued to increase gradually up to 22 s. The elevated electrodermal activity after each stimulus was followed by a gradual decrease to baseline.

The electrodermal activity increased during all inspirations, the 1st deep inspiration responses seemed to be larger than the 2nd and 3rd. However, the mean scores for electrodermal activity during inspirations were not significantly different for both experimental days [*F*_day1(1.038, 12.452)_ = 0.165 and *F*_day2(1.709, 12.000)_ = 1.024, *p* >> 0.05].

Table 1 shows that these electrodermal activity changes upon stimuli are highly significant, except inspirations on day 2. The table also shows that there were no significant differences in electrodermal activity responses between the 2 days for each of the five skin areas (*p* >> 0.05) for all 13 subjects. In the resting supine position, there were no fluctuations in the electrodermal activity, whereas spontaneous fluctuations were shown in both palms after position change (at ca 750s, 880s, 1,040s, and 1,140s), lasting to the start of the 1st inspiration. The steady state electrodermal activity in the palms are persistently increased upon position change.

Figure S1 shows that there was a strong correlation of electrodermal activity between the left and right palm for both experimental days. The calculated correlation coefficient was 0.992 for Day 1 and 0.991 for Day 2, and was obtained by taking the average electrodermal activity value for every second for both palms on the 2 days for all 13 subjects during the entire experiment.

### Cardiovascular Variables

Figure 1 shows simultaneous beat-to-beat recordings of mean arterial pressure (MAP), heart rate (HR), radial artery blood flow (RAF), skin perfusion in the palm (LDF-p), and dorsum (LDF-d) of the hand and electrodermal activity from five different skin areas from one subject. Whereas, electrodermal activity increased rapidly during each stimulus, there was a simultaneous abrupt fall in radial artery blood flow and skin perfusion in the palms.

**Figure 3:**
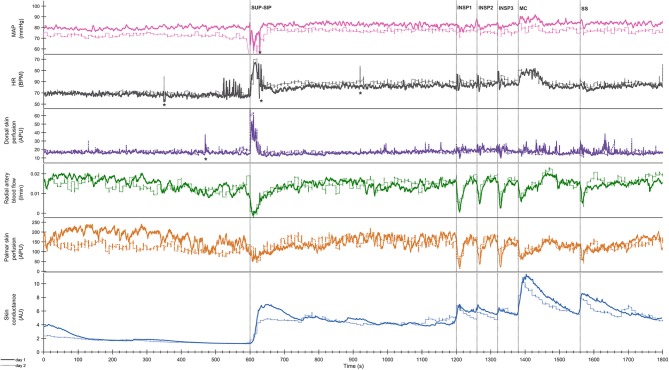
Shows mean of responses of MAP, HR, skin perfusion of palm (LDF-p) and dorsum (LDF-d) of the hand, radial artery blood flow (RAF), and electrodermal activity from the right palm from 13 subjects marked with solid colored lines for the entire 1st experimental day, and dashed black lines for the 2nd day. X-axis is time in seconds and the y-axis is in mmHg for MAP, BPM for HR, L/min for radial artery blood flow, arbitrary perfusion units (APU) of laser Doppler in palm and dorsum hand and arbitrary units of skin conductance in right palm, respectively. Vertical dashed lines mark the start of different stimuli corresponding to position change from supine (SUP) to sitting (SIP) (SUP-SIP), three deep inspirations (INSP1-3), mental challenge (MC) and a sound stimulus (SS). Several spikes (marked with *) just before and after position change, was caused by disturbances in the signal due to movement.

**Figure 4:**
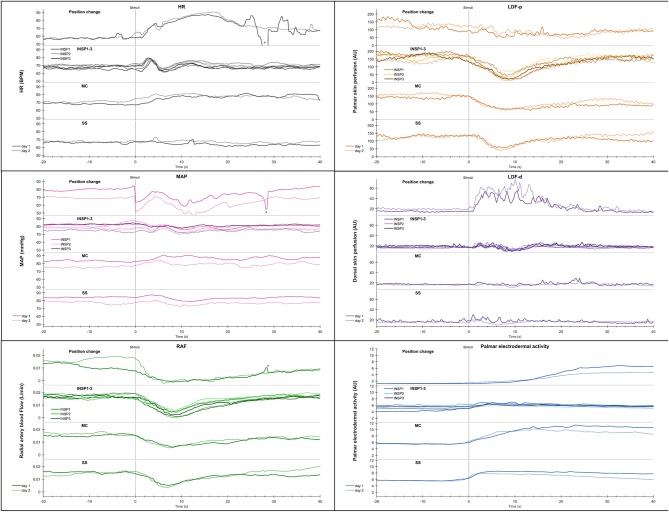
Shows mean of responses of HR, MAP, radial artery blood flow (RAF), skin perfusion of palm (LDF-p) and dorsum (LDF-d) of the hand, and electrodermal activity from the right palm from 13 subjects in an expanded time scale taken from Figure 3. The figure text details are identical to that in Figure 3.

Figure 3 shows mean recordings from 13 subjects of MAP, HR, radial artery blood flow, skin perfusion in the palm and dorsum of the hand and palm electrodermal activity for both experimental days (the electrodermal activity recordings are the same data as those in Figure 2). There was a very reproducible pattern for all subjects in cardiovascular variables between the 2 experimental days. There was no statistically significant differences for cardiovascular variables and palm electrodermal activity between the two experimental days for any stimulus. These calculations were based upon recordings from all 13 subjects during the time periods marked with gray boxes A–H in Figure 2. The relationship between electrodermal activity and cardiovascular variables upon the various stimuli are shown in greater detail in Figure 4. The figure shows that RAF (radial artery blood flow) and LDF-p (skin perfusion in the palms) decreases rapidly upon all stimuli: position change, inspiration (INSP1-3), mental challenge (MC), and sound stimulus (SS). Electrodermal activity variations are mainly synchronous and increases rapidly and simultaneously with an abrupt fall in RAF and LDF-p during each stimulus. Statistical analyses are shown in Table 1 (electrodermal activity) and Table S1 (cardiovascular variables).

Statistical analyses of the cardiovascular variables and electrodermal activity data shows a close correlation for position change, mental challenge and sound stimuli. In Figure S2 the correlation between radial artery blood flow and electrodermal activity is shown for position change and the corresponding correlation upon mental challenge in Figure S3.

Both in the resting supine and sitting positions, we observed spontaneous and synchronous variations in palmar skin perfusion, with similar variations in dorsal skin perfusion with a lower amplitude. HR also showed similar spontaneous variations in the resting situation. The steady state HR and MAP showed a persistent small increase upon position change. However, Figures 1, 2 show almost no spontaneous variations in electrodermal activity in the supine position but a few in the sitting position.

## Discussion

Our main new result was the pattern and reproducibility of the rapid and large changes in electrodermal activity in parallel to changes in acral blood flow, skin perfusion, HR, and MAP in response to minor physiological and mental stimuli in healthy subjects in the thermoneutral zone. The electrodermal activity patterns were almost identical in both palms and the responses are highly reproducible between two different experimental days. Some variations between individuals could be seen, but a main response pattern was seen in a majority of the subjects. These patterns of electrodermal activity recorded simultaneously with cardiovascular variables have not been shown before. Synchronous electrodermal activity was observed in the 1970's by Ogawa et al. (10–12, 29), followed by other authors as Ohhashi (24, 30), Machado-Moreira and Taylor (25), and Tronstad et al. (31).

The electrodermal activity response was large after position change, mental challenge and sound stimulus (Table 1). At position change, electrodermal activity started to increase some seconds after the given stimulus and peaked around half a minute later. This might be explained by the fact that the position change is a combined physical and mental stimulus and therefore might elicit a more complex and stronger physiological response than the other stimuli. Mental challenge and sound stimulus are mainly psychological stimuli. Several studies have also shown increased electrodermal activity to various mental and physiological stimuli (10, 12, 22–25, 30, 32–34).

The response pattern was highly reproducible between days, and although the response seemed to be higher upon the 1st inspiration, there was no significant difference between the repeated measure between 1st and 3rd inspiration [*F*_day1(1.038, 12.452)_ = 0.165 and *F*_(1.709, 12.000)_ = 1.024). In contrast, Ohhashi et al. did observe habituation upon stimuli as deep inspiration in their experiment (24).

A close correlation between the responses in radial artery blood flow and skin perfusion in the palms indicates a synchronous activation of the sympathetic efferent nerve fibers supplying the skin blood vessels, in particular the AVAs. The simultaneous increases in electrodermal activity in the palms upon each stimulus (Figures 1, 4) can be explained by a central mechanism controlling sympathetic nerve fibers innervating the sweat glands in the palms. In contrast, the skin perfusion measured in the dorsum of the hand as well as the electrodermal pattern from the face, back, and abdomen, did not seem to follow the rapid responses in electrodermal activity and skin perfusion in the palms. The dorsal skin is lacking AVAs thus it seems that the sympathetic innervation of AVAs and hand sweat glands is centrally controlled separately from skin perfusion and sweat glands elsewhere. We suggest that these findings may be a basis for the design of a reproducible protocol to be used for diagnostic studies and clinical evaluation of patients with hyperhidrosis. Importantly, the combination of electrodermal recordings, skin perfusion, and cardiovascular variables may reveal disturbances in these relationships in such patients. In our study, we show that in normal subjects no responses are seen from sweat glands in the face, back, and abdomen. This might be different in patients with hyperhidrosis, both before treatment and possibly after treatment with side effects of compensatory sweat responses.

During supine and sitting rest, numerous synchronous spontaneous fluctuations in radial artery blood flow and in palmar skin perfusion are clearly seen (Figure 1). These spontaneous fluctuations are individual and are therefore not seen in Figure 3 after averaging over all 13 subjects. In the supine position, there are almost no spontaneous variations in electrodermal activity, hence no sympathetic activity in the nerves innervating the sweat glands. On the contrary, the sympathetic activity innervating the skin vessels is highly activated, causing several spontaneous fluctuations within seconds. This may be explained by selective control of activity in the sympathetic nerves to skin vessels and sweat glands. In the sitting position, the electrodermal variations is seen with spontaneous fluctuations. There was, however, no co-variation between the spontaneous cardiovascular variables and electrodermal activity in the resting supine and sitting positions.

The spontaneous variations in palmar skin blood flow and the relationship to these cardiovascular variables has been described before (14, 17, 19, 26, 35). However, the lack of spontaneous changes in electrodermal activity in the resting supine position has previously not been shown.

### Limitations

The present study has some limitations. A stable ventilated room for temperature and humidity, but no regular climate chamber was used. Our study was based on recordings of electrodermal activity in healthy subjects due to emotional sweating, not thermoregulation, where these parameters are important in data presentation. Secondly, menstruation cycles among women was not taken into consideration. The strength of single one hand clap produced a sound of approximately 90 dB, but might have had some variations between the experiments. The subject underwent experiments both in the morning and afternoon. The protocol was tested in pilot experiments. Only after aggregated responses it was revealed that the electrodermal responses triggered by deep breathing or mental stress did not recover completely before the next challenge. In retrospect, the time allowed for recover between stimuli should have been increased.

## Conclusion

In conclusion, healthy subjects in the thermoneutral zone, showed almost identical patterns of electrodermal activity measured non-invasively in both palms, with rapid and large responses to minor physiological and mental stimuli, synchronous with fluctuations in radial artery blood flow and skin perfusion and closely connected to variations in blood pressure and heart rate. This may be explained by synchronous activation of sympathetic nerves. From our result, we suggest that the sympathetic innervation of AVAs and hand sweat glands is centrally controlled separately from skin perfusion and sweat glands elsewhere. The responses are reproducible from day to day, making it a stable and constant stimulus to be used for studies in patients with hyperhidrosis.

## New and Noteworthy

In humans exposed to minor physiological and mental stimuli, electrodermal activity increased rapidly within seconds and synchronously in both palms, in close relation to simultaneous skin perfusion and cardiovascular changes and with a high reproducibility. These results reveal reproducible, specific, and finely tuned autonomic control of sweat glands, heart, and blood vessels.

## Data Availability Statement

All datasets generated for this study are included in the article/Supplementary Material.

## Ethics Statement

The studies involving human participants were reviewed and approved by University of Oslo and Akershus University Hospital. The patients/participants provided their written informed consent to participate in this study.

## Author Contributions

JW and KT conceived and designed the research and edited and revised the manuscript. AH performed the experiments, prepared figures, and wrote the manuscript. KT and AH analyzed the data. JW, KT, and AH interpreted results of experiments and approved final version of the manuscript.

### Conflict of Interest

The authors declare that the research was conducted in the absence of any commercial or financial relationships that could be construed as a potential conflict of interest.

## Supplementary Material

Figure S1 The figure shows a scatterplot of electrodermal activity of the left and right palm on day 1 (blue circle) and day 2 (red square). The x-axis shows left palmar hand skin conductance in arbitrary units, while the y-axis shows right hand skin conductance in arbitrary units. The plot is based on a 30 min continuous recording of the entire experimental session, where each dot represents the average electrodermal activity value for every second from the right and left palm, respectively, as shown in Figure 2. The correlation coefficient was 0.992 for Day 1 and 0.991 for Day 2.

Figure S2 The figure shows a scatterplot of electrodermal activity of the right palm vs. against radial artery blood flow on day 1 (blue circle) and day 2 (red square). The x-axis shows right palmar hand skin conductance in arbitrary units, while the y-axis shows radial artery blood flow in L/min. The plot is based on a 10 s recording after position change from supine to sitting, where each dot represents the average value for every second from the right palm and radial artery blood flow as shown in Figure 3. The correlation coefficient was −0.783 for Day 1 and −0.932 for Day 2.

Figure S3 The figure shows a scatterplot of electrodermal activity of the right palm vs. against radial artery blood flow on day 1 (blue circle) and day 2 (red square). The figure text details are identical with to those in Figure S2, except that the recording is after mental challenge. The correlation coefficient was −0.918 for Day 1 and −0.984 for Day 2.

Table S1 Mean values of cardiovascular variables. Mean values, SEM and 95 % confidence interval (CI) from HR, MAP, RAF, LDF-p, and LDP-d from thirteen subjects upon different stimuli as position change from supine (SUP) to sitting (SIP) position (SIP_2min_–SUP_5min_ during 2 min and SIP_5min_–SUP_5min_ during 5 min), INSP1 (1st inspiration during 1 min), INSP2 (2nd inspiration during 1 min), INSP3 (3rd inspiration during 1 min), MC (mental challenge during 3 min) and SS (sound stimulus during 1 min) from the 1st and 2nd day and comparison between these 2 days. These mean values are calculated from the time periods indicated by the grey boxes marked with A-H shown in Figure 2. Supine position values were calculated as the average during a 5 min period (SUP_5min_) (A), while values in the sitting position was calculated 2 min after the position change (SIP_2min_) (B), and 5 min later in the experiment, which represents the baseline values in the sitting position (SIP_5min_) (C). The time period marked with the grey box C represents the baseline for the 1st, 2nd, and 3rd inspirations (INSP1, INSP2, INSP3), mental challenge (MC) and sound stimulus (SS). Electrodermal activity during inspiration (three times) and sound stimulus were calculated as the average 1 min after each stimulus, whereas 3 min after mental challenge. Values are estimated paired *t*-test mean and 95% confidence intervals for the group of thirteen healthy subjects. ^*^Significantly different from baseline (*P* < 0.05 for two-tailed test).

## Abbreviations

HR: Heart rate
MC: Mental challenge
INSP1-3: Inspiration 1st, 2nd, and 3rd
RAF: Radial artery blood flow
LDF-d: Dorsal skin perfusion
SIP: Sitting position
LDF-p: Palmar skin perfusion
SS: Sound stimulus
MAP: Middle artery blood pressure
SUP: Supine position.
