# Supplementary figures and images for: Rapid, Large, and Synchronous Sweat and Cardiovascular Responses Upon Minor Stimuli in Healthy Subjects. Dynamics and Reproducibility
Source: Front Neurol. 2020 Feb 4;11:51. doi: 10.3389/fneur.2020.00051 (PMC7010925; doi:10.3389/fneur.2020.00051)

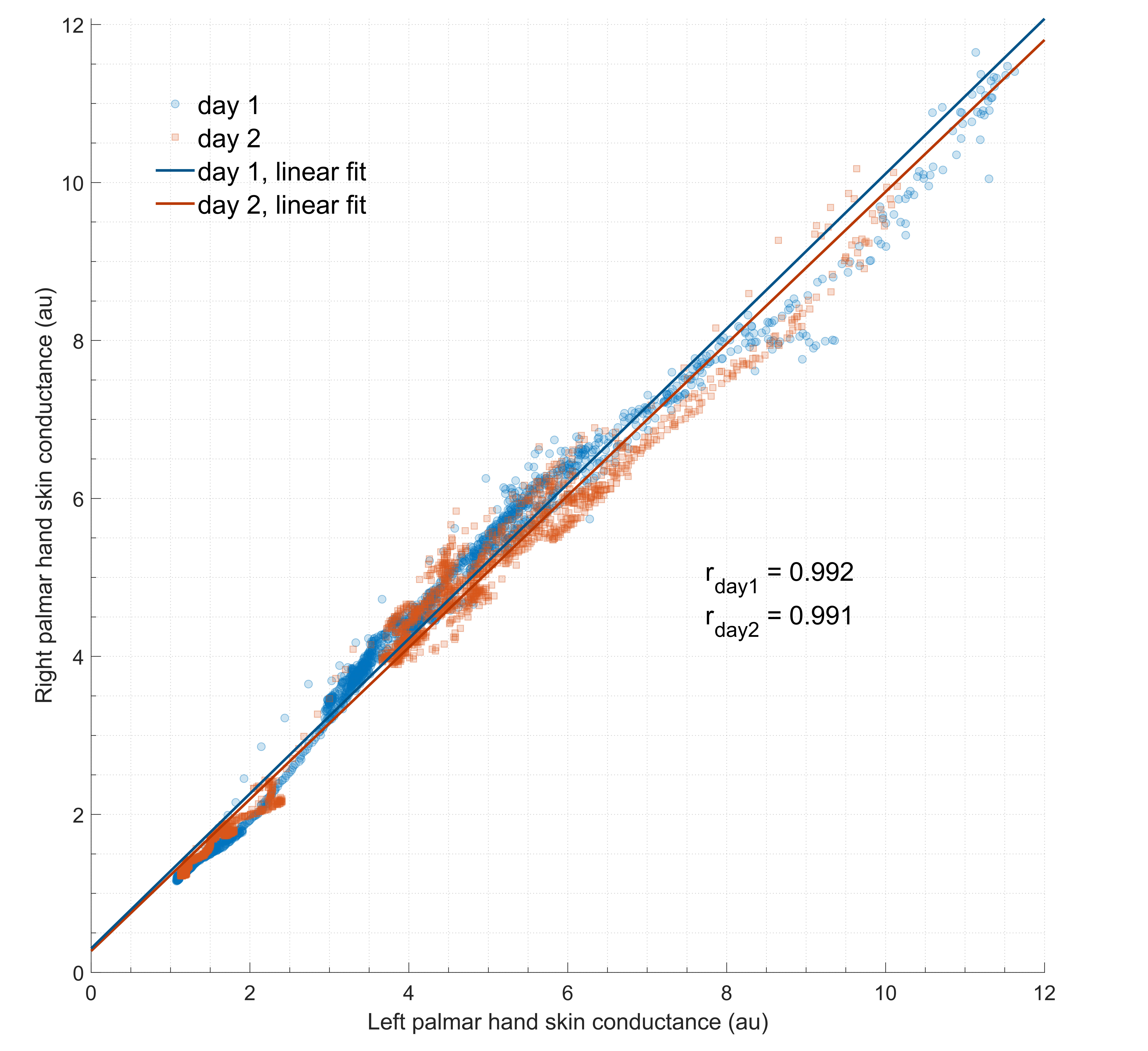

Supplement: Figure S1 — The figure shows a scatterplot of electrodermal activity of the left and right palm on day 1 (blue circle) and day 2 (red square). The x-axis shows left palmar hand skin conductance in arbitrary units, while the y-axis shows right hand skin conductance in arbitrary units. The plot is based on a 30 min continuous recording of the entire experimental session, where each dot represents the average electrodermal activity value for every second from the right and left palm, respectively, as shown in Figure 2. The correlation coefficient was 0.992 for Day 1 and 0.991 for Day 2. [file Image_1.JPEG]

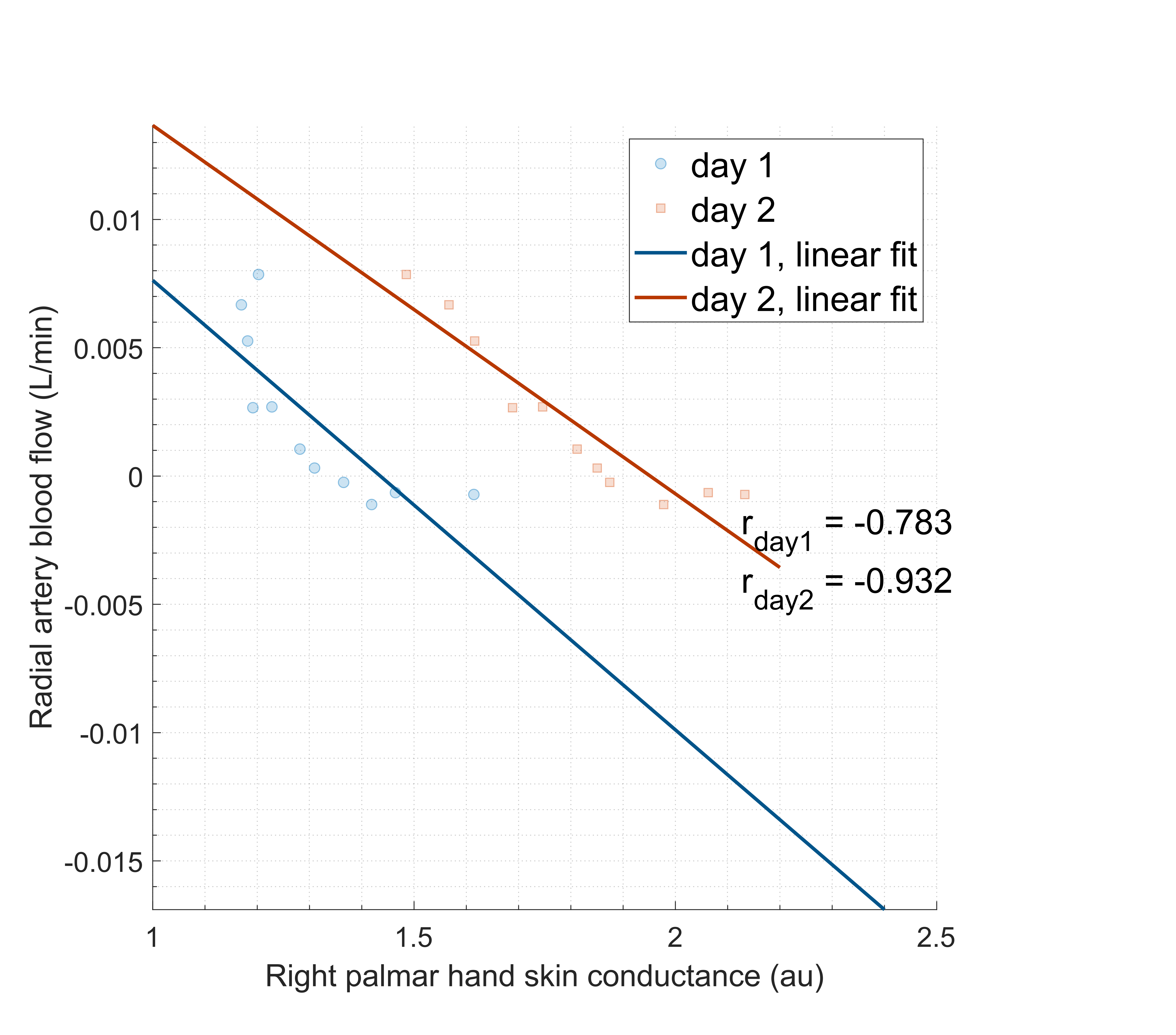

Supplement: Figure S2 — The figure shows a scatterplot of electrodermal activity of the right palm vs. against radial artery blood flow on day 1 (blue circle) and day 2 (red square). The x-axis shows right palmar hand skin conductance in arbitrary units, while the y-axis shows radial artery blood flow in L/min. The plot is based on a 10 s recording after position change from supine to sitting, where each dot represents the average value for every second from the right palm and radial artery blood flow as shown in Figure 3. The correlation coefficient was −0.783 for Day 1 and −0.932 for Day 2. [file Image_2.JPEG]

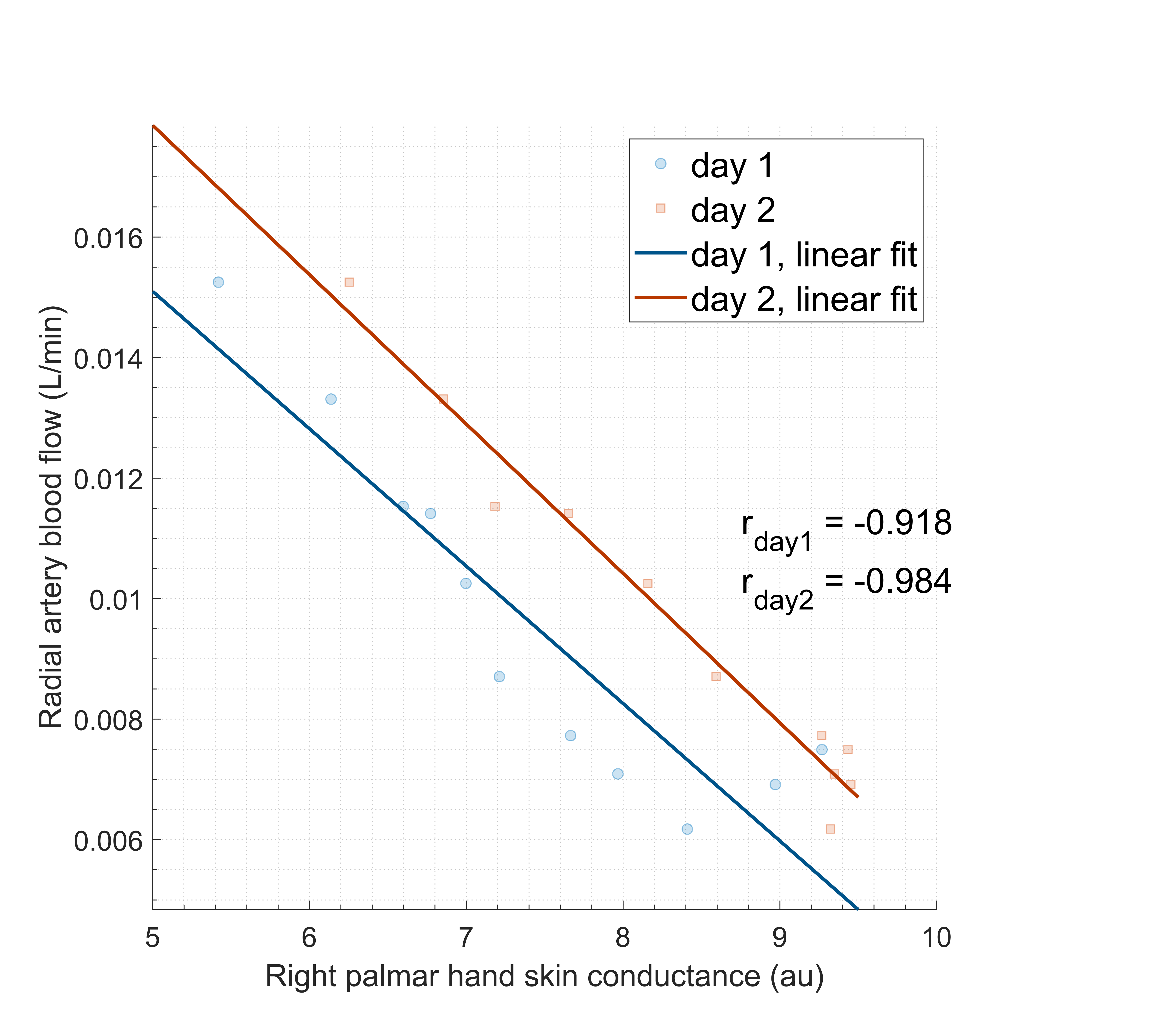

Supplement: Figure S3 — The figure shows a scatterplot of electrodermal activity of the right palm vs. against radial artery blood flow on day 1 (blue circle) and day 2 (red square). The figure text details are identical with to those in Figure S2, except that the recording is after mental challenge. The correlation coefficient was −0.918 for Day 1 and −0.984 for Day 2. [file Image_3.JPEG]
