# Supplementary material for: Rapid, Large, and Synchronous Sweat and Cardiovascular Responses Upon Minor Stimuli in Healthy Subjects. Dynamics and Reproducibility
Source: Front Neurol. 2020 Feb 4;11:51. doi: 10.3389/fneur.2020.00051 (PMC7010925; doi:10.3389/fneur.2020.00051)
Supplement: Table S1 — Mean values of cardiovascular variables. Mean values, SEM and 95 % confidence interval (CI) from HR, MAP, RAF, LDF-p, and LDP-d from thirteen subjects upon different stimuli as position change from supine (SUP) to sitting (SIP) position (SIP2min–SUP5min during 2 min and SIP5min–SUP5min during 5 min), INSP1 (1st inspiration during 1 min), INSP2 (2nd inspiration during 1 min), INSP3 (3rd inspiration during 1 min), MC (mental challenge during 3 min) and SS (sound stimulus during 1 min) from the 1st and 2nd day and comparison between these 2 days. These mean values are calculated from the time periods indicated by the grey boxes marked with A-H shown in Figure 2. Supine position values were calculated as the average during a 5 min period (SUP5min) (A), while values in the sitting position was calculated 2 min after the position change (SIP2min) (B), and 5 min later in the experiment, which represents the baseline values in the sitting position (SIP5min) (C). The time period marked with the grey box C represents the baseline for the 1st, 2nd, and 3rd inspirations (INSP1, INSP2, INSP3), mental challenge (MC) and sound stimulus (SS). Electrodermal activity during inspiration (three times) and sound stimulus were calculated as the average 1 min after each stimulus, whereas 3 min after mental challenge. Values are estimated paired t-test mean and 95% confidence intervals for the group of thirteen healthy subjects. *Significantly different from baseline (P < 0.05 for two-tailed test). [file Data_Sheet_1.PDF]

Day 1

**SIP$_{2min}$ – SUP$_{5min}$**	**Mean**	**SEM**	**95 % CI**	**p-value (2-tailed)**
**HR**	8.05	1.84	4.04-12.06	0.001*
**MAP**	21.22	2.02	16.83-25.61	0.000*
**RAF**	-0.004	0.001	-0.006-(-0.001)	0.005*
**LDF-p**	-57.71	37.18	-138.73-23.29	0.147
**LDF-d**	1.81	1.57	-1.61-5.23	0.271

**SIP$_{5min}$ – SUP$_{5min}$**	**Mean**	**SEM**	**95 % CI**	**p-value (2-tailed)**
**HR**	7.87	1.88	3.78-11.97	0.001*
**MAP**	24.29	1.72	20.54-28.04	0.000*
**RAF**	0.001	0.002	-0.003-0.005	0.581
**LDF-p**	4.32	18.42	-35.81-44.45	0.818
**LDF-d**	0.82	2.95	-5.60-7.24	0.786

**INSP1 - SIP$_{5min}$**	**Mean**	**SEM**	**95 % CI**	**p-value (2-tailed)**
**HR**	0.69	0.97	-1.42-2.81	0.488
**MAP**	-2.11	0.96	-4.20-(-0.02)	0.048*
**RAF**	-0.003	0.002	-0.008-0.002	0.251
**LDF-p**	-53.34	38.02	-136.18-29.5	0.186
**LDF-d**	0.99	0.90	-0.97-2.94	0.294

**INSP2 - SIP$_{5min}$**	**Mean**	**SEM**	**95 % CI**	**p-value (2-tailed)**
**HR**	1.50	1.06	-0.81-3.81	0.184
**MAP**	-1.11	0.85	-2.96-0.74	0.216
**RAF**	-0.001	0.001	-0.003-0.002	0.513
**LDF-p**	-48.41	35.06	-124.80-27.99	0.193
**LDF-d**	2.15	1.18	-0.42-4.72	0.094

**INSP3 - SIP$_{5min}$**	**Mean**	**SEM**	**95 % CI**	**p-value (2-tailed)**
**HR**	2.18	0.77	0.51-3.86	0.015*
**MAP**	-0.97	0.96	-3.06-1.12	0.333
**RAF**	-0.002	0.002	-0.007-0.002	0.290
**LDF-p**	-58.15	41.21	-147.92-31.63	0.184
**LDF-d**	0.86	0.74	-0.76-2.47	0.270

MC_{3min} - SIP_{5min}	Mean	SEM	95 % CI	p-value (2-tailed)
**HR**	3.95	1.67	0.32-7.59	0.035*
**MAP**	2.55	1.64	-1.01-6.11	0.145
**RAF**	-0.000	0.001	-0.003-0.002	0.829
**LDF-p**	-59.46	47.56	-163.09-44.18	0.235
**LDF-d**	-0.26	0.82	-2.05-1.52	0.752

SS_{1min} - SIP_{5min}	Mean	SEM	95 % CI	p-value (2-tailed)
**HR**	-1.97	1.37	-4.96-1.01	0.175
**MAP**	1.00	1.75	-2.81-4.81	0.578
**RAF**	-0.003	0.002	-0.007-0.001	0.096
**LDF-p**	-76.48	43.70	-171.69-18.73	0.106
**LDF-d**	-0.77	0.82	-2.55-1.00	0.362

Day 2

**SIP$_{2min}$ – SUP$_{5min}$**	**Mean**	**SEM**	**95 % CI**	**p-value (2-tailed)**
**HR**	11.12	1.87	7.05-15.20	0.000*
**MAP**	21.72	2.14	17.06-26.38	0.000*
**RAF**	-0.003	0.003	-0.009-0.003	0.343
**LDF-p**	9.32	30.35	-56.80-75.44	0.764
**LDF-d**	4.68	1.59	1.21-8.15	0.012*

**SIP$_{5min}$ – SUP$_{5min}$**	**Mean**	**SEM**	**95 % CI**	**p-value (2-tailed)**
**HR**	10.12	1.65	6.53-13.73	0.000*
**MAP**	26.74	2.03	22.32-31.16	0.000*
**RAF**	0.003	0.002	-0.003-0.008	0.295
**LDF-p**	46.52	30.71	-20.38-113.43	0.156
**LDF-d**	-0.71	1.04	-2.98-1.55	0.504

**INSP1 - SIP$_{5min}$**	**Mean**	**SEM**	**95 % CI**	**p-value (2-tailed)**
**HR**	1.11	0.52	-0.01-2.25	0.052
**MAP**	-1.54	0.91	-3.52-0.44	0.115
**RAF**	-0.002	0.001	-0.004-0.0004	0.099
**LDF-p**	-8.47	9.35	-28.84-11.90	0.383
**LDF-d**	1.39	0.64	-0.00-2.77	0.050

**INSP2 - SIP$_{5min}$**	**Mean**	**SEM**	**95 % CI**	**p-value (2-tailed)**
**HR**	1.23	0.70	-0.29-2.75	0.104
**MAP**	-2.42	1.07	-4.75-(-0.10)	0.042*
**RAF**	0.001	0.001	-0.001-0.002	0.554
**LDF-p**	14.59	15.46	-19.09-48.27	0.364
**LDF-d**	2.42	0.88	0.50-4.34	0.018*

**INSP3 - SIP$_{5min}$**	**Mean**	**SEM**	**95 % CI**	**p-value (2-tailed)**
**HR**	2.61	0.80	0.87-4.34	0.007*
**MAP**	-2.61	1.26	-5.36-0.14	0.061
**RAF**	-0.001	0.001	-0.003-0.001	0.211
**LDF-p**	-3.36	17.19	-40.81-34.09	0.848
**LDF-d**	1.09	1.19	-1.51-3.68	0.380

**MC_{3min} - SIP_{5min}**	**Mean**	**SEM**	**95 % CI**	**p-value (2-tailed)**
**HR**	2.13	1.00	-0.04-4.31	0.054
**MAP**	0.49	1.35	-2.45-3.44	0.721
**RAF**	-0.000	0.001	-0.003-0.003	0.980
**LDF-p**	-13.47	8.89	-32.85-5.90	0.156
**LDF-d**	0.31	1.04	-1.95-2.57	0.770

**SS_{1min} - SIP_{5min}**	**Mean**	**SEM**	**95 % CI**	**p-value (2-tailed)**
**HR**	-1.68	0.74	-3.29-(-0.07)	0.042*
**MAP**	-0.82	1.28	-3.62-1.98	0.535
**RAF**	-0.001	0.001	-0.004-0.002	0.621
**LDF-p**	-21.53	14.30	-52.69-9.64	0.158
**LDF-d**	0.46	1.40	-2.59-3.50	0.750

Day 1 vs Day 2

SUP - SIP	Mean	SEM	95 % CI	p-value (2-tailed)
**HR**	-3.22	2.16	-7.93-1.49	0.162
**MAP**	7.13	4.07	-1.74-16.01	0.105
**RAF**	-0.001	0.002	-0.005-0.004	0.848
**LDF-p**	-18.15	29.12	-81.60-45.29	0.545
**LDF-d**	-3.87	2.26	-8.79-1.05	0.112

INSP1	Mean	SEM	95 % CI	p-value (2-tailed)
**HR**	-3.30	1.52	-6.61-0.02	0.051
**MAP**	4.44	2.85	-1.76-10.64	0.145
**RAF**	-0.001	0.002	-0.006-0.003	0.615
**LDF-p**	-3.66	20.69	-48.73-41.41	0.862
**LDF-d**	1.83	3.97	-6.82-10.49	0.653

INSP2	Mean	SEM	95 % CI	p-value (2-tailed)
**HR**	-2.61	1.42	-5.71-0.50	0.092
**MAP**	6.32	3.07	-0.37-13.00	0.062
**RAF**	-0.002	0.003	-0.008-0.005	0.621
**LDF-p**	-21.79	29.85	-86.84-43.25	0.479
**LDF-d**	1.97	4.48	-7.79-11.72	0.668

INSP3	Mean	SEM	95 % CI	p-value (2-tailed)
**HR**	-3.30	1.87	-7.38-0.78	0.103
**MAP**	6.64	3.18	-0.29-13.58	0.059
**RAF**	-0.001	0.002	-0.006-0.004	0.563
**LDF-p**	-13.58	29.01	-76.78-49.62	0.648
**LDF-d**	2.01	3.92	-6.54-10.55	0.618

MC	Mean	SEM	95 % CI	p-value (2-tailed)
**HR**	-1.06	1.32	-3.93-1.82	0.439
**MAP**	7.06	3.31	-0.15-14.27	0.054
**RAF**	-0.0004	0.003	-0.007-0.006	0.896
**LDF-p**	-4.78	24.15	-57.39-47.83	0.846
**LDF-d**	1.66	3.02	-4.93-8.25	0.593

SS	Mean	SEM	95 % CI	p-value (2-tailed)
**HR**	-3.17	1.58	-6.60-0.27	0.068
**MAP**	6.83	13.12	-1.10-14.75	0.085
**RAF**	-0.002	0.003	-0.009-0.004	0.408
**LDF-p**	-13.75	17.18	-51.19-23.69	0.439
**LDF-d**	1.00	3.39	-6.38-8.39	0.772
